# Inhibition of K_Ca_2 and K_v_11.1 Channels in Pigs With Left Ventricular Dysfunction

**DOI:** 10.3389/fphar.2020.00556

**Published:** 2020-05-06

**Authors:** Carlotta Citerni, Jeppe Kirchhoff, Lisbeth Høier Olsen, Stefan Michael Sattler, Morten Grunnet, Nils Edvardsson, Bo Hjorth Bentzen, Jonas Goldin Diness

**Affiliations:** ^1^Biomedical Institute, University of Copenhagen, Copenhagen, Denmark; ^2^Acesion Pharma, Copenhagen, Denmark; ^3^Department of Veterinary Disease Biology, University of Copenhagen, Frederiksberg, Denmark; ^4^Department of Cardiology, Heart Center, Copenhagen University Hospital, Rigshospitalet, Copenhagen, Denmark; ^5^Department of Molecular and Clinical Medicine, Sahlgrenska Academy at Sahlgrenska University Hospital, Gothenburg, Sweden

**Keywords:** ventricular dysfunction, KCa2 channels, small-conductance Ca2+-activated K+ channels, atrial fibrillation, pig model, dofetilide

## Abstract

**Background:**

Inhibition of K_Ca_2 channels, conducting I_KCa_, can convert atrial fibrillation (AF) to sinus rhythm and protect against its induction. I_KCa_ inhibition has been shown to possess functional atrial selectivity with minor effects on ventricles. Under pathophysiological conditions with ventricular remodeling, however, inhibiting I_KCa_ can exhibit both proarrhythmic and antiarrhythmic ventricular effects. The aim of this study was to evaluate the effects of the I_KCa_ inhibitor AP14145, when given before or after the I_Kr_ blocker dofetilide, on cardiac function and ventricular proarrhythmia markers in pigs with or without left ventricular dysfunction (LVD).

**Methods:**

Landrace pigs were randomized into an AF group (n = 6) and two control groups: SHAM1 (n = 8) and SHAM2 (n = 4). AF pigs were atrially tachypaced (A-TP) for 43 ± 4 days until sustained AF and LVD developed. A-TP and SHAM1 pigs received 20 mg/kg AP14145 followed by 100 µg/kg dofetilide whereas SHAM2 pigs received the same drugs in the opposite order. Proarrhythmic markers such as short-term variability of QT (STV_QT_) and RR (STV_RR_) intervals, and the number of premature ventricular complexes (PVCs) were measured at baseline and after administration of drugs. The influence on cardiac function was assessed by measuring cardiac output, stroke volume, and relevant echocardiographic parameters.

**Results:**

I_KCa_ inhibition by AP14145 did not increase STV_QT_ or STV_RR_ in any of the pigs. I_Kr_ inhibition by dofetilide markedly increased STV_QT_ in the A-TP pigs, but not in SHAM operated pigs. Upon infusion of AP14145 the number of PVCs decreased or remained unchanged both when AP14145 was infused after baseline and after dofetilide. Conversely, the number of PVCs increased or remained unchanged upon dofetilide infusion. Neither AP14145 nor dofetilide affected relevant echocardiographic parameters, cardiac output, or stroke volume in any of the groups.

**Conclusion:**

I_KCa_ inhibition with AP14145 was not proarrhythmic in healthy pigs, or in the presence of LVD resulting from A-TP. In pigs already challenged with 100 µg/kg dofetilide there were no signs of proarrhythmia when 20 mg/kg AP14145 were infused. K_Ca_2 channel inhibition did not affect cardiac function, implying that K_Ca_2 inhibitors can be administered safely also in the presence of LV dysfunction.

## Introduction

Heart failure and atrial fibrillation (AF) are present concomitantly in many patients and can cause and exacerbate each other through structural cardiac remodeling, activation of neurohormonal mechanisms, and rate-related impairment of left ventricular function.

Efficacy of class III antiarrhythmic drugs relies on the ability to prolong the atrial effective refractory period (AERP). Traditional class III drugs such as dofetilide are inhibitors of the potassium current carried by the K_v_11.1 protein, I_Kr_. Since I_Kr_ is present in the ventricles, inhibition of the current can delay ventricular repolarization seen as an increased QT interval on the surface electrocardiogram (ECG). Prolongation of the QT interval corrected for heart rate (QTc) is associated with increased risk of the potentially lethal ventricular arrhythmia torsades de pointes (TdP) (Workman et al., 2011) and is commonly used as a surrogate marker for proarrhythmicity. However, QTc prolongation alone is an imperfect proarrhythmia marker since not all drugs that prolong the QTc are proarrhythmic (Piccini et al., 2009). Another surrogate marker for proarrhythmicity that has been suggested to be superior to QTc interval prolongation to predict TdP-risk is beat-to-beat variability quantified by short-term variability of the QT interval (Thomsen et al., 2004, Varkevisser et al., 2012).

The K_Ca_2 channels, also known as small conductance Ca^2+^- activated K^+^, or small-conductance Ca2+-activated K+ (SK) channels, conducting I_KCa_ are a relatively novel target for AF treatment. I_KCa_ inhibition with different compounds can convert AF to sinus rhythm and/or protected against its induction. This has been shown in AF models in isolated perfused hearts from rat, guinea pig, and rabbit as well as in *in vivo* models in rats, dogs, pigs, goats, and horses (Diness et al., 2010; Diness et al., 2011; Skibsbye et al., 2011; Qi et al., 2013; Haugaard et al., 2015; Diness et al., 2017; Gatta et al., 2019). I_KCa_ inhibition has been shown to possess functional atrial selectivity with minor effects on ventricles (Diness et al., ; Diness et al., 2010; Qi et al., 2013; Skibsbye et al., 2014; Kirchhoff et al., 2019).

Under certain pathophysiological conditions such as myocardial infarction and congestive heart failure ventricular K_Ca_2 current can be increased. Blocking K_Ca_2 currents under such circumstances can exhibit both neutral (Lubberding et al., 2019), proarrhythmic (Chang et al., 2013; Bonilla et al., 2014), and antiarrhythmic (Chua et al., 2011; Gui et al., 2013; Hundahl et al., 2017; Yin et al., 2017; Lubberding et al., 2019) ventricular effects, depending on the experimental setup.

We hypothesized that inhibiting K_Ca_2 channels would increase ventricular proarrhythmia markers in pigs with ventricular structural remodeling and dysfunction while pigs without structural remodeling will not be affected. The aim of this study was to evaluate the effects of the K_Ca_2 channel inhibitor AP14145, when given before or after the K_v_11.1 channel blocker dofetilide, on cardiac function and ventricular proarrhythmia markers in pigs with or without atrial and ventricular structural remodeling and dysfunction.

## Materials and Methods

### Experimental Animals

All animal studies were performed under a license from the Danish Ministry of Environment and Food (license No. 2014-15-0201-00390), in accordance with the Danish guidelines for animal experiments according to the European Commission Directive 86/609/EEC. A total of 18 female Danish landrace pigs were included in the study. The pigs were 11 weeks old on the day of arrival and weighed 25–35 kg. The pigs were randomized into two groups: long-term atrially tachypaced (A-TP) pigs with AF and sham operated control (SHAM) pigs. The A-TP pigs were tachypaced until AF that was resistant to cardioversion by 4 mg/kg vernakalant (18 ± 2 days of A-TP) as previously described (Diness et al., 2017) and then for additionally 25 ± 4 days for a total of 43 ± 4 days of A-TP. All pigs were treated with a daily dose of 250 µg digoxin in order to prevent clinical signs of heart failure, but developed some degree of left ventricular dysfunction as described previously (Citerni et al., 2020). The surgical procedure for pacemaker implantation and post-operative treatments were previously described (Diness et al., 2017).

**Figure 1:**
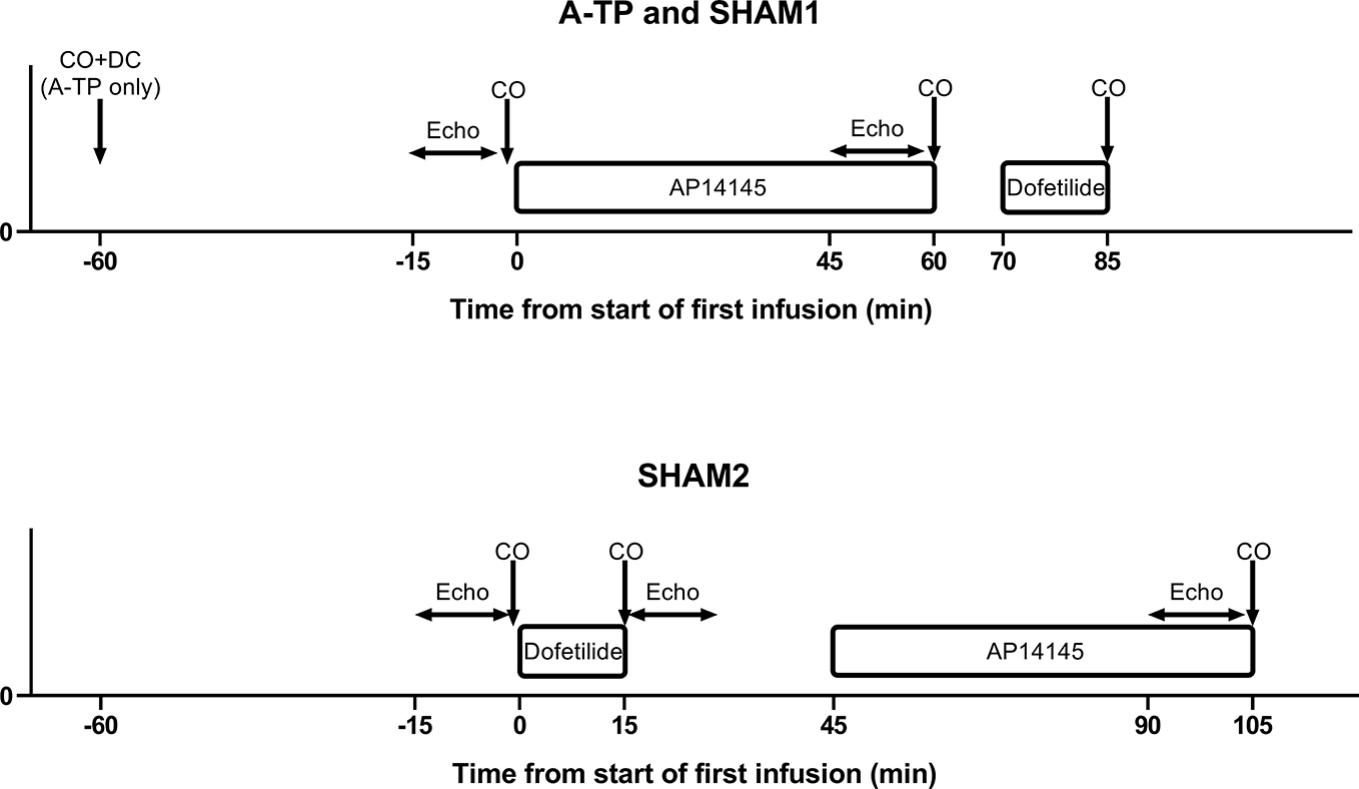
Timelines for the atrially tachypaced (A-TP), SHAM1, and SHAM2 groups. DC, direct current cardioversion; CO, cardiac output measurement; Echo, echocardiography; AP14145, a dose of 20 mg/kg AP14145 infused over 60 min; Dofetilide, a dose of 0.1 mg/kg dofetilide infused over 15 min.

All pigs underwent a terminal experiment in which the effects of K_Ca_2 channel inhibition by AP14145 and K_v_11.1 channel inhibition with dofetilide on cardiac output (CO) and different echocardiographic and electrophysiological parameters were tested. The timelines of the terminal experiments with A-TP, SHAM1, and SHAM2 pigs can be observed in **Figure 1**. The pigs were divided as follows: 12 control animals (4 SHAM1 and 8 SHAM2 pigs) and 6 A-TP. One of the A-TP pigs was excluded from CO and stroke volume (SV) statistical analyses due to spontaneous conversion to sinus rhythm (SR) upon induction of anesthesia.

After premedication with Zoletil pig mixture (see **Supplementary Materials** for details), pigs were anesthetized with intravenous propofol and fentanyl (15 mg/kg/h and 5 µg/kg/h, respectively) and intubated and ventilated with a tidal volume of 10 ml/kg and a respiration frequency of 12–14/min. During surgery partial pressure of carbon dioxide (PaCO_2_), blood pressure and ECG were monitored and the pig was given 6 ml/kg/h isotonic salt solution. CO was measured by thermodilution method *via* a Swan-Ganz catheter placed into the pulmonary artery as previously described (Sattler et al., 2019). The external mammary vein was catheterized for drug infusion.

### Drugs

In the present study, A-TP pigs received a 1-h infusion of 20 mg/kg AP14145 60 min after electrical cardioversion. Ten minutes after the end of AP14145 infusion, 0.1 mg/kg dofetilide was infused over 15 min. SHAM1 pigs followed the same infusion protocol as the A-TP pigs. SHAM2 pigs received dofetilide 60 min after induction of anesthesia followed by AP14145 30 min after the end of dofetilide injection. The longer break between the infusions in the SHAM2 pigs was necessary to allow echocardiographic assessment after dofetilide infusion.

### Electrophysiological Assessment of Proarrhythmia Risk

Drug-induced prolongation of the QTc interval is often considered an important safety variable, and if excessive it may be a predictor of TdP (Belardinelli et al., 2003). However, QTc prolongation alone is an imperfect proarrhythmia marker since drugs that prolong the QTc interval to the same extent do not necessarily carry the same risk of proarrhythmia (Belardinelli et al., 2003; Piccini et al., 2009). The beat-to-beat variability of repolarization is quantified by short-term variability (STV). STV of QT intervals (STV_QT_) has been suggested to be superior to QTc prolongation in predicting drug-induced TdP risk of various cardiovascular drugs in certain experimental assays (Thomsen et al., 2004; Detre et al., 2005). In this study changes in both the QTc (corrected for heart rate with Bazett’s formula) and STV_QT_ were analyzed to assess the risk of ventricular proarrhythmia. Pigs were equipped with three bipolar standard ECG leads. The ECGs analyzes was carried out semi-automatically with manual adjudication in LabChart. The STV_QT_, the STV of RR intervals (STV_RR_), and QTc were calculated from 30 consecutive beats just before and at the end of each compound infusion using the following formula: STV= ∑|D_n+1_–D_n_|/[30*√2], where D represents the duration of RR or QT and 30 is the number of consecutive beats chosen for the measurement (Thomsen et al., 2004).

In this study, premature ventricular complexes (PVCs) were calculated by manual adjudication from bipolar standards leads over a 15-min period just before (baseline) as well as immediately after infusion of AP14145 and dofetilide, respectively.

### Assessment of Cardiac Function

#### Echocardiography

Echocardiography was performed before and during the last 15 min of AP14145 infusion in order to examine effects on inotropy and other parameters for cardiac function (**Figure 1**). In SHAM2 pigs, echocardiograms were also obtained after infusion of dofetilide. A standardized transthoracic echocardiographic examination (TTE) together with the newly introduced trans-diaphragmatic echocardiographic (TDE) approach (Citerni et al.,) with the pig in dorsal recumbency using right parasternal and left apical windows was performed and digitally stored using an Philips iE33 machine (Philips Healthcare, Amsterdam, the Netherlands) equipped with a S5-1 transducer (3.5 MHz) and continuous electrocardiographic (ECG) monitoring. Offline echocardiographic analysis was performed using EchoPAC version 113 with the observer blinded to the identity of the recording. All reported values are averages of three consecutive heart cycles during sinus rhythm.

Left ventricular (LV) function was assessed as LV ejection fraction (LVEF) with the four chamber modified Simpson’s method of disks and LV fraction area change (FAC) calculated by the ratio between maximum diastolic and systolic LV internal areas measured in short axis (SAX) (Thomas et al., 1993; Ravn et al., 2014). Left atrial (LA) volume was evaluated with the biplane modified Simpson’s method of disks (Lang et al., 2015), using the four chamber view (van den Dorpel et al., 2018).

Furthermore LV diastolic function was assessed by mitral valve inflow Doppler patterns [transmitral E/A ratio and E velocity deceleration time (EDT)] and pulse wave tissue Doppler imaging (E’ septal and E/E’ ratio) (van den Dorpel et al., 2018). Peak aorta flow velocity was estimate using pulse wave Doppler imaging (Ravn et al., 2014).

#### Estimation of Cardiac Output and Stroke Volume

CO and SV were measured by thermo dilution *via* the Swan-Ganz catheter using a rapid injection of 10 ml of saline (0.9%) injection at 4°C into the pulmonary artery *via* the jugular vein. The CO was calculated as an average of three consecutive measurements in a row at four times points for the A-TP and at 3 time points for the SHAM pigs: immediately before ECV in AF only for A-TP; 1 h after ECV in SR just before AP14145 infusion for A-TP and 1 h after anesthesia for SHAM; immediately after AP14145 and dofetilide infusions (**Figure 1**). SV was calculated dividing the CO with the HR.

### Statistical Analyses

GraphPad Prism 8 and LabChart 7 were used for data analysis and figures.

STV_QT_ and STV_RR_ data were analyzed as follows: outliers were removed with the ROUT (1%) test. Repeated measures analyses were made with ANOVA if there were no missing values and with mixed effects model in the case of missing values (e.g., due to removal of outliers). Differences between STV_QT_ and STV_RR_ between start and end of infusion of either AP14145 or dofetilide were then compared using Sidak’s adjustment for multiple comparisons. QTcB (The QT interval corrected for heart rate with Bazett's formula) and RR interval data were analyzed in the same way. The number of PVCs was analyzed with a Friedman test followed by Dunn’s multiple comparisons test using the number of PVCs after the first infusion of test compound as the control. Cardiac output (CO) and stroke volume (SV) data were analyzed as follows: repeated measures analyses were made with ANOVA. The CO and SV data were analyzed with one-way ANOVA followed by Sidak’s multiple comparisons test using the data after the first infusion of test compound as the control.

All echocardiographic data were tested for normality with D’Agostino-Pearson test. Data that followed a Gaussian distribution were analyzed with Student T-test, and the rest of the data were tested with the Wilcoxon signed-rank test. Outliers were removed with the ROUT (1%) test. Wilcoxon signed rank test was used to compare echocardiographic data before and after K_Ca_2 channel inhibition in the SHAM1 pigs. In the SHAM2 pigs, one-way ANOVA was used.

Data are summarized using the mean ± SEM. P-values are given with three decimals and considered statistically significant if <0.05.

## Results

At the time of the pacemaker implantation, the pigs had an average body weight of 31 ± 1 and 32 ± 1 kg for A-TP and SHAM, respectively. At follow-up after 43 ± 4 days of A-TP the weight had increased to 71 ± 4 and 69 ± 4 kg, respectively. No significant difference in body weight between the groups was observed at any point.

### Substrate Development

The heart rate (HR) of the A-TP pigs was 130 ± 14 bpm during AF, which upon electrical cardioversion (ECV) decreased to 71 ± 3 bpm which was similar to the HR of SHAM pigs at follow up after 43 ± 4 days of A-TP (74 ± 4 bpm). The A-TP pigs had LV and LA dysfunction and structural remodeling characterized by increased abundance of extracellular matrix (ECM) and enlarged LV and LA with reduced contractility compared the SHAM controls.

After 43 ± 4 days of A-TP, A-TP pigs had developed reduced myocardial contractility documented by LV systolic dysfunction with reduced LVEF and FAC. Among diastolic function parameters, LA volume was found increased, E/A was increased by 50%, and peak A decreased by 21% even though non-significantly, as well as five out of six pigs developed mild mitral valve regurgitation compared to control. Furthermore, A-TP pigs showed structural remodeling characterized by increased abundance of extracellular matrix (ECM) in all cardiac chambers. By contrast, all the echocardiographic parameters and histological analyses were within normality in all SHAM pigs as previously documented (Citerni et al., ).

### Effects on STV_QT_ and STV_RR_

**Figure 2:**
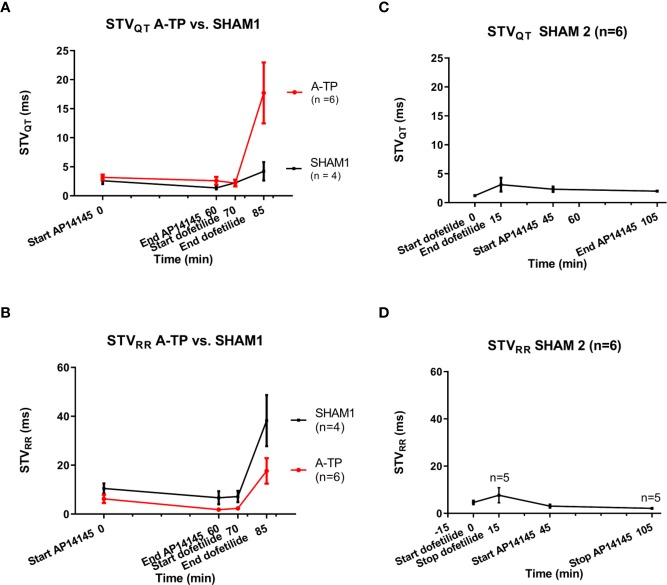
STV_QT_ and STV_RR_ before and after infusion of AP14145 and dofetilide. Left column **(A, B)**: STV_QT+RR_ from the atrially tachypaced (A-TP) pigs and the SHAM1 pigs. Right column **(C**, **D)**: STV_QT+RR_ from the SHAM2 pigs. Two outlier values were removed from the SHAM2 STV_RR_ group.

The STV_QT_ and STV_RR_ values before and after infusion of AP14145 and dofetilide are given in **Table 1** and **Figure 2**. K_Ca_2 channel inhibition by AP14145 did not increase STV_QT_ or STV_RR_ from baseline values in A-TP and SHAM1 pigs. The same was the case when AP14145 was applied in the presence of dofetilide in the SHAM2 pigs. On the contrary, a small and not statistically significant decrease of STV_QT_ after AP14145 administration could be observed in all groups of pigs.

**Table 1:** STV_QT_ and STV_RR_ expressed in milliseconds (ms) before and after infusion of AP14145 and dofetilide.

Group	Parameter	Start AP14145	End AP14145	Adjusted p-value	Start dofetilide	End dofetilide	Adjusted p-value
**ATP**	STV_QT_	3.2 ± 0.5	2.6 ± 0.7	0.810	2.2 ± 0.6	17.7 ± 5.2	0.045
	STV_RR_	6.2 ± 1.7	1.8 ± 0.4	0.093	2.3 ± 0.5	17.7 ± 5.2	0.081
**SHAM1**	STV_QT_	2.6 ± 0.6	1.4 ± 0.2	0.136	2.2 ± 0.3	4.2 ± 1.6	0.544
	STV_RR_	10.5 ± 2.1	6.7 ± 2.7	0.275	7.2 ± 2.3	38.2 ± 10.5	0.177
**SHAM2**	STV_QT_	3.0 ± 0.8	2.1 ± 0.2	0.328	4.6 ± 0.9	7.7 ± 3.2	0.747
	STV_RR_	3.1 ± 0.9	2.1 ± 3.2	0.607	4.6 ± 0.8	7.7 ± 0.2	0.432

K_v_11.1 inhibition by dofetilide markedly increased STV_QT_ in the A-TP pigs and, to a lesser and not statistically significant extent, in both groups of SHAM operated pigs.

A-TP and SHAM1 pigs received AP14145 followed by dofetilide with a 10 min interval. In the SHAM2 group the pigs received dofetilide first, and AP14145 afterwards with a 30 min interval. Repeated measures analyses were made with ANOVA for all groups except for the SHAM2 group where a mixed model analysis was applied due to removal of two outlier values. Differences between STV_QT_ and STV_RR_ between start and end of infusion of either AP14145 or dofetilide were compared using Sidak’s adjustment for multiple comparisons.

### Effects on QTcB and Heart Rate

**Figure 3:**
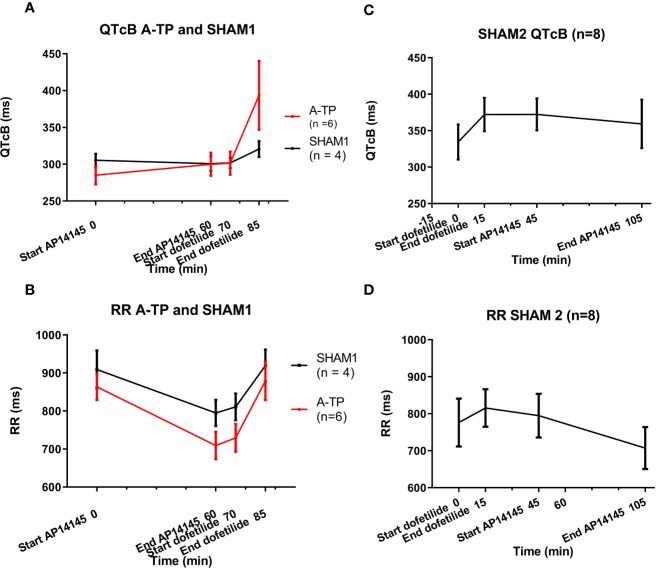
QTcB and RR intervals. Left column **(A**, **B)**: QTcB and RR intervals from the atrially tachypaced (A-TP) pigs and the SHAM1 pigs. Right column **(C, D)**: QTcB and RR intervals from the SHAM2 pigs.

The QTcB and RR interval values before and after infusion of AP14145 an dofetilide are given in **Table 2** and **Figure 3**. The QTcB was not increased significantly by AP14145 in any of the groups. However, the heart rate was increased (seen as a drop in RR intervals) in all three groups, although only to a statistically significant extent in the SHAM1 group. Conversely, dofetilide decreased the heart rate in all groups of pigs, but only to a statistically significant extent in the A-TP group. As expected, dofetilide increased the QTcB in all groups of pigs, although only to a statistically significant extent in the SHAM2 group.

**Table 2:** QTcB and RR intervals (in ms) before and after infusion of AP14145 and dofetilide.

Group	Parameter	Start AP14145	End AP14145	Adjusted p-value	Start dofetilide	End dofetilide	Adjusted p-value
**A-TP**	QTcB	285 ± 13	300 ± 16	0.327	301 ± 16	393 ± 47	0.115
	RR	863 ± 35	709 ± 36	0.095	729 ± 37	879 ± 50	0.001
**SHAM1**	QTcB	305 ± 9	301 ± 10	0.799	302 ± 7	321 ± 11	0.113
	RR	909 ± 50	795 ± 34	0.045	811 ± 35	919 ± 42	0.073
**SHAM2**	QTcB	372 + 22	359 + 33	0.694	334 ± 24	372 ± 23	<0.001
	RR	795 + 59	707 + 57	0.325	776 ± 65	816 ± 51	0.373

A-TP and SHAM1 pigs received AP14145 followed by dofetilide. In the SHAM2 group the pigs received dofetilide first, and AP14145 afterwards. Repeated measures analyses were made with ANOVA for all groups. Differences between start and end of infusion of either AP14145 or dofetilide were compared using Sidak’s adjustment for multiple comparisons.

### Effects on the Number of Premature Ventricular Complexes

**Figure 4:**
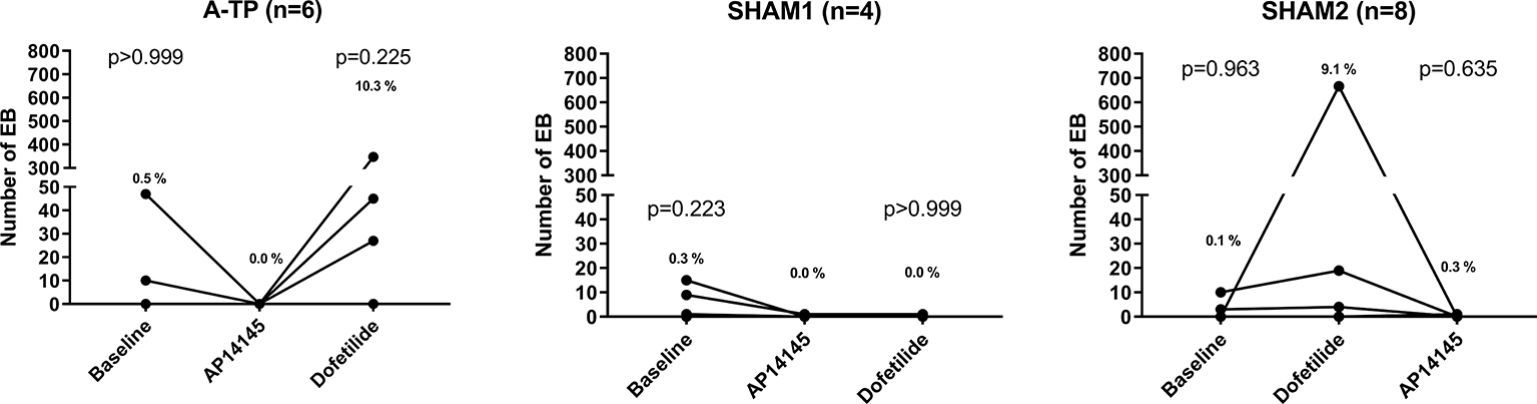
The number of premature ventricular complexes (PVCs) during the 15 min preceding the first infusion of test compound and after the end of AP14145 and dofetilide infusions, respectively. Left: the atrially tachypaced (A-TP) group. Middle: the SHAM1 group. Right: the SHAM2 group. The percentages of PVCs out of the total number of heartbeats within each period are also given in the graphs. Data were analyzed with a Friedman test followed by Dunn’s multiple comparisons test using the number of PVCs after the first infusion of test compound as the control. The adjusted p-values in the graphs are from Dunn’s test.

Upon infusion of AP14145 the number of PVCs decreased or remained unchanged both when AP14145 was infused after baseline and after dofetilide (**Figure 4**). Conversely, the number of PVCs increased or remained unchanged upon dofetilide infusion both when it was infused after baseline and after AP14145. However, none of these changes were statistically significant.

### Effects of K_Ca_2 and K_v_11.1 Inhibition on Impaired and Normal Cardiac Function

In the A-TP pigs with impaired cardiac function as well as in the healthy SHAM1 pigs neither K_Ca_2 nor K_v_11.1 inhibition exerted significant effect on echocardiographic variables when AP14145 was given before dofetilide during the terminal experiment (**Table 3**).

**Table 3:** Measurements of systolic and diastolic myocardial functions.

	SHAM1 Baseline	SHAM1 AP14145	P value	A-TP Baseline	A-TP AP14145	P value
Ejection fraction; EF (%)	66 ± 3	66 ± 3	>0.999	49 ± 5^8^	44 ± 3^8^	0.392
Fractional area change; FAC (%)	66 ± 4	59 ± 9	0.625	40 ± 4^6^	37 ± 3^6^	0.552
Peak aortic flow velocity; (m/s)	1.3 ± 0.1	1.2 ± 0.1	0.625	1.1 ± 0.1^8^	1.0 ± 0.1^8^	0.244
Peak E (m/s)	0.68 ± 0.03	0.58 ± 0.02	0.125	0.63 ± 0.05^7^	0.54 ± 0.03^7^	0.117
Peak A (m/s)	0.60 ± 0.06	0.78 ± 0.05	0.125	0.50 ± 0.06^7^	0.55 ± 0.07^7^	0.297
EDT (ms)	206 ± 22	192 ± 15	0.625	152 ± 6^7^	170 ± 15^7^	0.121
E/A	1.17 ± 0.13	0.76 ± 0.03	0.125	1.40 ± 0.26^7^	1.10 ± 0.12^7^	0.159
E’ septal (m/s)*	0.10 ± 0.01	0.09 ± 0.01	0.250	0.08 ± 0.01^8^	0.07 ± 0.01^8^	0.504
E/E’ septal	6.7 ± 0.4	7.1 ± 0.7	0.625	8.4 ± 0.6^7^	9.2 ± 1.5^7^	0.691
LA end-diastolic Vol. (ml)	28 ± 3	24 ± 1	0.625	54 ± 8^7^	58 ± 7^7^	0.718
LA end-systolic Vol. (ml)	11 ± 1	8 ± 1	0.625	38 ± 7^7^	35 ± 5^7^	0.718

Changes from before to after infusion of the KCa2 inhibitor AP14145 in the A-TP and SHAM l groups. All pigs were in sinus rhythm. Volumes of LA and LV measured with SMOD method in left apical 4 chamber view; LV area in systole and diastole measured in SAX view using 2D echocardiography to calculate fractional area change (FAC). Peak aorta flow velocity was estimated using pulse wave Doppler imaging. MV Peak E, early diastolic velocity; MV Peak A, atrial contraction velocity; EDT, transmitral flow deceleration time; E/A, peak E and A ratio; E' septal, isovolumic relaxation time; E/E', peak E and isovolumic relaxation time ratio. Superscripts are the number of animals from which the median in question has been calculated in case of missing observations. Values are expressed in mean ± SEM. P values are expressed in the right columns (paired Student t-test for the A-TP pigs and Wilcoxon test for the SHAM1 pigs). *E' lateral was not possible to measure at the baseline, therefore this parameter has been excluded from the analysis.

In the healthy SHAM2 pigs, K_v_11.1 inhibition by dofetilide did not impact echocardiographic parameters when given before AP14145. On the other hand, K_Ca_2 inhibition by AP14145 did lower the peak aorta velocity (1.4 ± 0.1 vs. 1.0 ± 0.1 m/s, P=0.045) compared to dofetilide and increased the E/E’ septal ratio (7.6 ± 0.5 *vs.* 11 ± 0.3, P=0.040) when AP14145 was applied after dofetilide (**Table 4**).

**Table 4:** Measurements of systolic and diastolic myocardial function before and after infusion of 0.1 mg/kg of dofetilide over 15 min, and after infusion of 20 mg/kg AP14145 over 60 min.

	SHAM2 before dofetilide	SHAM2 after dofetilide	P value	SHAM2 after AP14145	P value
Ejection fraction; EF (%)	69 ± 3^7^	69 ± 3^7^	0.989	66 ± 2^7^	0.526
Fractional area change; FAC (%)	63 ± 2^5^	57 ± 2^5^	0.152	51 ± 2^5^	0.126
Peak aortic flow velocity; (m/s)	1.4 ± 0.1^7^	1.4 ± 0.1^7^	0.970	1.0 ± 0.1^7^	0.045
Peak E (m/s)	0.75 ± 0.08^3^	0.78 ± 0.03^3^	0.845	0.70 ± 0.08^3^	0.403
Peak A (m/s) *	0.65 ± 0.10 ^3^	0.75 ± 0.04^3^	>0.999	0.79 ± 0.06^3^	>0.999
EDT (ms) *	139 ± 24^3^	144 ± 6^3^	>0.999	142 ± 19^3^	0.828
E/A *	1.2 ± 0.3^3^	1.1 ± 0.1^3^	>0.999	0.9 ± 0.1^3^	0.205
E’ septal (m/s)	0.08 ± 0.01	0.09 ± 0.01	0.447	0.07 ± 0.01	0.258
E/E’ septal	11 ± 1^3^	7.6 ± 1^3^	0.278	11 ± 1^3^	0.040
LA end-diastolic Vol. (ml)	29 ± 2^7^	28 ± 1^7^	0.889	26 ± 2^7^	0.499
LA end-systolic Vol. (ml)	12 ± 1^7^	12 ± 1^7^	0.999	9 ± 1^7^	0.073

Values are expressed in mean ± SEM. Superscripts are the number of animals from which the median in question has been calculated in case of missing observations. Values are expressed in mean ± SEM. *Friedman test was performed for these parameters. Parameters were compared as follows: before vs. after administration of dofetilide and after dofetilide vs. after AP14145.

**Figure 5:**
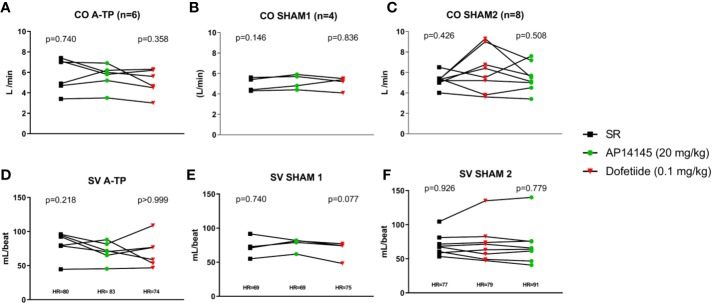
Top: cardiac output (CO) at baseline [60 min after cardioversion in the atrially tachypaced (A-TP) pigs] and immediately after infusion of each compound in the A-TP **(A)**, the SHAM1 **(B)**, and SHAM2 **(C)** groups. Bottom: stroke volume (SV) at baseline and immediately after infusion of each compound in the A-TP **(D)**, the SHAM1 **(E)**, and SHAM2 **(F)** groups. Repeated measures analyses were made with ANOVA in all groups. The data after the first infusion of test compound were used as the control.

CO was recorded during SR 1 h after electrical cardioversion for A-TP and 1 h after anesthesia for SHAM, and again immediately after AP14145 and dofetilide infusions (**Figure 1**). Neither AP14145 nor dofetilide affected CO or SV in any of the groups (**Figure 5**).

## Discussion

In the present study we evaluated the effects of the K_Ca_2 channel inhibitor AP14145, when given before or after the K_v_11.1 channel blocker dofetilide, on cardiac function and ventricular proarrhythmia markers in pigs with or without atrial and ventricular structural remodeling and dysfunction. K_Ca_2 channel inhibition by AP14145 neither showed any signs of ventricular proarrhythmia nor did it negatively affect cardiac function.

### Previous Studies With AP14145

Previously, four studies including AP14145 have been published (Diness et al., 2017; Simó-Vicens et al., 2017; Kirchhoff et al., 2019; Lubberding et al., 2019). In 2017, our group published the first study describing how AP14145 acts as a negative allosteric modulator of the K_Ca_2 channel by right-shifting the curve of activation by calcium (Simó-Vicens et al., 2017). Also, in 2017 we published another study describing how AP14145 *in vitro* is selective for K_Ca_2 channels compared to other relevant cardiac ion channels (Diness et al., 2017). In the same study, we demonstrated that *in vivo* in pigs, AP14145 had functional selectivity for atria versus ventricles and that it converted AF that was resistant to treatment with vernakalant to sinus rhythm. In 2019, we published a study showing that K_Ca_2 channel inhibition by AP14145 was functionally atrial selective compared with K_v_11.1 inhibition by dofetilide and ondansetron which was functionally selective for ventricles (Kirchhoff et al., 2019). Later in 2019 a group in close collaboration with ours, found that inhibition of K_Ca_2 channels with AP14145 was neither beneficial nor detrimental to ventricular arrhythmia development in a porcine model of acute myocardial infarction (Lubberding et al., 2019).

### AP14145 and Dofetilide Doses

In previous experiments with AP14145 conversion of vernakalant-resistant AF occurred already at a dose level of 5 mg/kg and thus an infusion of 20 mg/kg as was used in the current study would be expected to give exposure well-above a level that would be relevant for conversion of AF (Diness et al., 2017). A relatively high dose of dofetilide, 0.1 mg/kg was used as a positive control for increasing proarrhythmia markers. In the clinic, cardioversion of AF is usually attempted with ~10 (4–12) µg/kg of dofetilide and thus a ten-fold higher dose was expected to be proarrhythmic (Sedgwick et al., 1995; Falk et al., 1997; Nørgaard et al., 1999; Lindeboom et al., 2000).

### Effects of K_Ca_2 Channel Inhibition on Ventricular Electrophysiology

The effects of K_Ca_2 channel inhibition with AP14145 on ventricular electrophysiology were neutral or slightly antiarrhythmic, seen as a small and not statistically significant decrease of the STV_QT_ in all groups of pigs as well as an unchanged or decreased number of PVCs upon infusion of AP14145. The QTcB was not affected to any significant extent after AP14145 infusion in any of the pigs, similar to what has previously been reported in pigs (Diness et al., 2017). These effects of AP14145 on arrhythmia markers were the same in the presence of the K_v_11.1 inhibitor dofetilide as seen in the SHAM2 group: STV_QT_, QTcB, and the number of PVCs were all slightly decreased, but not to any statistically significant extent.

In contrast, the K_v_11.1 inhibitor dofetilide prolonged the QTcB and increased STV_QT_ in all groups of pigs (although the QTcB increase was only statistically significant in the SHAM2 pigs and the STV_QT_ increase was only statistically significant in the ATP pigs). The effects of dofetilide were comparable between the two SHAM groups where STV _QT_ and QTcB were increased to similar extents. Regarding the number of PVCs, it seemed that dofetilide alone increased the number of PVCs as seen in the SHAM2 group, whereas this was not the case in the SHAM1 group where AP14145 was present. In the A-TP pigs dofetilide seemed even more proarrhythmic; STV_QT_, QTcB, and the number of PVCs was increased markedly more than in either of the SHAM groups.

Based on our experiments we cannot conclude if AP14145 affected the proarrhythmic effect of dofetilide in A-TP pigs. However, suffice it to say, that there was no indication of an increase in any arrhythmia markers after the infusion of AP14145 in any of the groups. On the contrary, the effect of infusion of AP14145 on any of the measured proarrhythmia markers was neutral at worst.

### STV_QT_ as a Predictor of Torsades de Pointes

Hinterseer et al. conducted descriptive human studies demonstrating that STV_QT_ was approximately doubled in individuals at risk for repolarization-dependent arrhythmias compared to matched controls. This was the case in patients known to be at risk for drug-induced TdP (Hinterseer et al., 2008), patients with inherited long QT syndrome (LQTS) (Hinterseer et al., 2009), and in patients with documented non-ischaemic congestive heart failure (Hinterseer et al., 2010).

In dogs with chronic atrio-ventricular block (CAVB), the STV_QT_ and the STV of left ventricular monophasic action potential duration (STV_LV_) is superior to the QT interval in assessing the risk of drugs causing TdP (Varkevisser et al., 2012). In a study by Dunnink et al. the STV_LV_ increased from 1.4 ± 0.6 to 3.2 ± 1.6 ms and TdP occurred in 56% of the dogs after treatment with 25 µg/kg dofetilide (Dunnink et al., 2012). Similar a study by Thomsen et al. found that 25 µg/kg dofetilide increased the STV_LV_ from 2.3 ± 0.6 to 4.2 ± 1.5 ms and TdP occurred in 74% of the dogs (Thomsen et al., 2007).

The changes we observed in STV_QT_ after infusion of 100 µg/kg dofetilide in the SHAM pigs are of the same magnitude as were observed in the CAVB dogs in the two studies by Dunnink et al. and Thomsen et al. Interestingly, the increase observed in the A-TP pigs in our study (from 2.2 ± 0.6 to 17.7 ± 5.2 ms) is, to the best of our knowledge, much larger than any increase in STV_LV_ or STV_QT_ leading to TdP in the CAVB dog model after testing “unsafe” drugs such as sertindole, dofetilide, bepridil, terfenadine, and sotalol (Varkevisser et al., 2012). However, no single episode of TdP was observed in any of the pigs.

It therefore seems that pigs, even pigs with remodeled ventricles and very increased STV_QT_, are less susceptible to TdP than CAVB dogs. However, STV_QT_ might still be a valuable marker of proarrhythmia in pigs since it is increased after dofetilide infusion. The fact that STV_QT_ is increased to a greater extent by dofetilide in the A-TP pigs than in the SHAM pigs hints at a reduced repolarization reserve in the A-TP pigs.

### Effects on the RR Interval and the STV_RR_

In the SHAM1 pigs the RR interval decreases during AP14145 treatment and returns to baseline values during dofetilide infusion. The pattern is the same in the SHAM2 pigs: during dofetilide infusion the RR increases (although less pronounced than in the SHAM1 pigs) and decreases to values below baseline afterwards during AP14145 infusion. These observations fit very well with the half-lives of the drugs: the most-reported half-life in the literature is 10 h for dofetilide and the half-life of AP14145 has been reported to be very short—only 24 min (Diness et al., 2017; Ibrahim and Tivakaran, 2020). Thus, in the SHAM1 pigs during dofetilide infusion the decreasing effects of AP14145 on the RR interval are rapidly wearing off and this in combination with the effects of dofetilide gives an increased variability in the RR interval, and thus a rather steep rise in STV_RR_.

If the combination of AP14145 and dofetilide should generally increase STV_RR_, we would expect this to give an even more pronounced effect in the SHAM2 pigs at the end of AP14145 infusion where the plasma concentration of both compounds is high.

### Effects on Cardiac Function

The echocardiographic examination at the terminal experiment revealed that neither dofetilide nor AP14145 further worsened the already impaired cardiac function in the A-TP. Dofetilide and AP14145 also did not negatively affect normal cardiac function in the SHAM pigs.

Despite the higher E/E’ septal ratio after AP14145 compared to dofetilide in the SHAM2 pigs, both parameters values after AP14145 were in line with the baseline values before dofetilide infusion. Therefore, we do not think that these findings are of clinical importance.

The peak aortic velocity was unaffected by AP14145 when given alone in the SHAM1 and A-TP pigs. Similarly, the peak aortic velocity was unaffected by dofetilide when given alone in the SHAM2 pigs. When AP14145 was administered after dofetilide in the SHAM2 pigs the peak aortic velocity was slightly lower. This finding is probably of very limited clinical importance since it is within normal ranges for peak aortic velocity.

In line with the echocardiographic examination, the results from the measurements of the CO and SV demonstrated that neither dofetilide nor AP14145 affected any of these parameters.

## Limitations

This study was only powered to detect relatively large and uniform changes. Due to the relatively large variation in some of the parameters that were tested changes that could have been clinically relevant were not found to be statistically significant. Nevertheless, the data obtained sufficed to obtain meaningful insight into whether K_Ca_2 channel inhibition with AP14145 was proarrhythmic in a setting of LV dysfunction resulting from A-TP or after challenging SHAM pigs with infusion of a high dose dofetilide.

## Conclusion

K_Ca_2 channel inhibition with AP14145 was not proarrhythmic in healthy pigs, or in the presence of LV dysfunction resulting from A-TP. Also, when 20 mg/kg AP14145 were infused in pigs already challenged with 100 µg/kg dofetilide there were no signs of proarrhythmia. On the contrary, after AP14145 infusion the number of PVCs as well as the QTc and the STV_QT_ were all slightly decreased, although not to an extent that was statistically significant. Furthermore, K_Ca_2 channel inhibition did not affect cardiac function. This implies that K_Ca_2 inhibitors can be administered safely also in the presence of LV dysfunction.

## Data Availability Statement

The datasets generated for this study are available on request to the corresponding author.

## Ethics Statement

The animal study was reviewed and approved by Danish Ministry of Environment and Food.

## Author Contributions

JD, BB, MG, and CC contributed to the conception and design of the study. SS, LO, NE, and CC performed and/or helped with analysis of echocardiographic data. JD and CC wrote first draft of manuscript. All authors contributed to manuscript revision, read, and approved the submitted version.

## Funding

The study was supported by Innovation Fund Denmark, the Carlsberg Foundation, the Wellcome Trust (award reference no. 100406/Z/12/Z), and the European Union’s Horizon 2020 research and innovation programme under the Marie Skłodowska-Curie grant agreement no. 675351.

## Conflict of Interest

JD, MG, NE, and BB are fully or partly employed in Acesion Pharma and JD, MG, and BB are inventors of Acesion Pharma patents within the field of KCa2 channels. JK and CC are former employees of Acesion Pharma.

The remaining authors declare that the research was conducted in the absence of any commercial or financial relationships that could be construed as a potential conflict of interest.
