# Supplementary material for: Inhibition of KCa2 and Kv11.1 Channels in Pigs With Left Ventricular Dysfunction
Source: Front Pharmacol. 2020 May 6;11:556. doi: 10.3389/fphar.2020.00556 (PMC7219273; doi:10.3389/fphar.2020.00556)
Supplement: Supplementary file 1 [file DataSheet_1.docx]

# Supplementary data

Table 1: Equipment

| **Equipment** | **Producer** | **Model** | **Rationale for choosing this** |
| --- | --- | --- | --- |
| AF Neurostimulator | Medtronic, Dublin, Ireland | Synergy versitrel | Implantable stimulator capable of delivering sufficiently fast and long electrical impulses for cardiac tachypacing. |
| AF Neurostimulator | Medtronic, Dublin, Ireland | Itrel 3 | Implantable stimulator capable of delivering sufficiently fast and long electrical impulses for cardiac tachypacing. Slightly smaller than the synergy versitrel making it easier to implant. No longer in production. |
| Sterile filter | Thermo Scientific, Waltham, Massachusetts, USA | Nalgene, Rapid flow 90 mm filter unit, 250-500 ml. | State of the art product for sterile filtration. |
| Implantable leads | St Jude Medical, Little Canada, Minnesota, US | Tendril (2088T/58 cm) | State of the art electrical lead with active fixation. Used routinely for patients. |
| Televet, Holter monitor | Engel Engineering Service GmbH, Heusenstamm, Germany | Televet-100 | State of the art Telemetric ECG System for veterinary medicine. |
| Infusion pump | Shenzhen Shenke Medical Instrument Technical Development Co.,Ltd., Shenzhen P.R.China | SK-500I syringe pump | Syringe pump capable of delivering up to 500 ml/h. |
| 50 ml syringe | Medical Surgical Systems BD, Albertslund, Denmark | BD Plastipak 50 ml Luer-Lok syringe | Standard 50 ml Luer-Lok syringe compatible with most syringe pumps. |
| Patient Monitor | Agilent, Glostrup, Denmark, | Viridia | Monitoring of ECG, Blood pressure SP0_2_ and temperature of the pig during surgery. |
| Respirator | Demeca, Rødovre, Denmark | Siesta i TS | Respiration of the pig during surgery. 500 mL/min 0_2_ and 2500 mL/min air. |
| Infusion pump | B.Braun Melsungen AG,  Melsungen, Germany | Infusomat space | Pump for infusion of Propofol. |
| Infusion pump | B.Braun Melsungen AG, Melsungen, Germany | Perfuser compact | Syringe pump for infusion of fentanyl. |
| Infusion pump | Heska,  Loveland, Colorado, USA | Vet/IV | Infusion pump for saline solution. |
| Cardiovascular ultrasound machine | (Philips Health care, Amsterdam, The Netherlands) | iE33 machine equipped with an S5-1 transducer (3.5 MHz) | Ultrasound machine for echocardiographic images acquisition |

Table 2: Drugs

| **Drug** | **Producer** | **Strength** | **Dosing** | **Rationale** |
| --- | --- | --- | --- | --- |
| Zoletil pig mix | Unit for experimental medicine, Copenhagen University | Solution for injection, 250 mg dry tiletamin+zolazepam, 6.5 ml xylazine 20 mg/ml, 1.25 ml ketamine 100 mg/ml, 2.5 ml butorphanol 10 mg/ml, and 2 ml methadone 10 mg/ml | 0.1 ml/kg, IM | Used for pre-anaesthesia of the pigs. |
| Gentacoll® patches | Swedish Orphan Biovitrum | 32.5 mg gentamycin in 5x5 cm patch | 32.5 mg in implantation pocket. | Broad-spectrum antibiotic to avoid infection in the implantation pocket. |
| Propolipid | Fresenius Kabi | Liquid for infusion, emulsion of 10 mg/ml propofol. | 15 mg/kg/h, IV | Anaesthetic agent |
| Fentanyl | “B. Braun”,  “2Care4”, or “Sandoz” | Solution for injection, 50 µg/ml | 5 µg/kg/h | Opioid. Anaesthetic and analgesic agent. |
| Norostrep Vet. | ScanVet Animal Health A/S, Fredensborg | Liquid for infusion, emulsion of 200.000 IU (200 mg)/ml. | 2 ml on the pacemaker just before implantation. | Antibiotic for penicillin- and dihydrostreptomycin-vulnerable bacteria. |
| Curamox prolongatum Vet. | Boehringer Ingelheim | Liquid for infusion, emulsion of 150mg amoxicillin/ml | 15 mg/kg, IM | Broad-spectrum antibiotic for amoxicillin-vulnerable bacteria with prolonged effect (>48 h) |
| Metacam | Boehringer Ingelheim | 15 mg meloxicam/ml | Oral suspension  0.5 mg/kg PO daily for three days after implantation | NSAID for post operational pain. |
| Clamoxyl Vet. | Orion Pharma Animal Health A/S | 510 mg amoxicillin/g | Powder for oral solution 40 mg/kg PO daily for 5 days after implantation | Broad-spectrum antibiotic for amoxicillin-vulnerable bacteria. |

All drugs used in this study are commercially available and sourced from the open market within the European Union. The zoletil pig mix is not a marketed combination and was prepared by the Unit for Experimental Medicine at Copenhagen University.

Table 3: Software

| **Software** | **Supplier** | **Rationale** |
| --- | --- | --- |
| GraphPad Prism 8.2.0 | GraphPad Software, Inc. | Software for simple statistical analyses and for graphical representations of data. |
| LabChart 7.3.7. | ADInstruments | A platform for multiple recording devices to work together, allowing the acquisition of biological signals from multiple sources simultaneously. Allows for semi-automated analyses of ECGs. |
| Televet ECG software 6.0.0 | Engel Engineering Services GmbH, Germany | Works with the Televet100 Holter monitor for acquisition of ECGs from conscious pigs. |
| EchoPAC software | GE healthcare, USA | Software for echocardiographic images analyses |

**Post-operative treatment**

Postoperative analgesia was accomplished by administration of Meloxicam (Metacam, Boehringer Ingelheim, Germany) 0.5 mg/kg orally (PO) once daily for post-operatory pain. Amoxicillin (Clamoxyl vet 510 mg/g, Orion Pharma Animal Health A/S, Copenhagen, DK) , powder for oral solution, 40 mg/kg PO daily was given for 5 days after implantation to lower the risk of postoperative infections.

**Table 4: overview of detailed inclusion/exclusion of the pig population in the procedures described in the study.**

| Pig ID | **Experimental group** | **terminal experiment** | **Echo at implant** | **Echo after AP14145** | **Echo after dofetilide** | **Proarrhythmic biomarkers**  **(QTc, STV, PVCs)** | **CO & SV** |
| --- | --- | --- | --- | --- | --- | --- | --- |
| 283 | A-TP | YES | YES | YES | NO | YES | YES |
| 303 | A-TP | YES | NO | YES | NO | YES | YES |
| 380 | A-TP | YES | YES | YES | NO | YES | YES |
| 406 | A-TP | YES | YES | YES | NO | YES | YES |
| 146 | A-TP | YES | YES | YES | NO | YES | YES |
| 147 | A-TP | YES | YES | YES | NO | YES | YES |
| 1 | SHAM 1 | YES | NO | YES | NO | YES | YES |
| 33 | SHAM 1 | YES | NO | YES | NO | YES | YES |
| 34 | SHAM 1 | YES | NO | YES | NO | YES | YES |
| 99 | SHAM 1 | YES | YES | YES | NO | YES | YES |
| 2 | SHAM 2 | YES | YES | YES | YES | YES | YES |
| 8 | SHAM 2 | YES | NO | YES | YES | YES* | YES |
| 407 | SHAM 2 | YES | YES | YES | YES | YES | YES |
| 141 | SHAM 2 | YES | YES | YES | YES | YES* | YES |
| 142 | SHAM 2 | YES | YES | YES | YES | YES | YES |
| 153 | SHAM 2 | YES | YES | YES | YES | YES | YES |
| 189 | SHAM 2 | YES | YES | YES | YES | YES | YES |
| 190 | SHAM 2 | YES | YES | YES | YES | YES | YES |
| 191 | SHAM 2 | YES | YES | YES | YES | YES | YES |
| Number of pigs (n) |  | 6 A-TP  8 SHAM2  4 SHAM1 | 6 A-TP  8 SHAM2  4 SHAM1 | 6 A-TP  8 SHAM2  4 SHAM1 | 6 A-TP  8 SHAM2  4 SHAM1 | 6 A-TP  8 SHAM2  4 SHAM1 | 6 A-TP  8 SHAM2  4 SHAM1 |

Cardiac output = CO; Stroke volume = SV; QT interval corrected for the heart rate = QTc; short term variability of QT and RR intervals = STV_QT+RR_; premature ventricular complexes = PVCs

Pig #407 did complete the study but was not included in the statistical analyses because of pericarditis.

* Outliers removed from the STV_QT+RR_ data statistic.
